# Large language model chatbot-based text-to-SQL application for database analyses in liver diseases and hepatology research

**DOI:** 10.1093/jamiaopen/ooag106

**Published:** 2026-06-19

**Authors:** Aryana T Far, Steve Sun, Gabrielle Jutras, Melinda B Wang, Shohei Burns, Joseph Owens, Victor Galvez, Ma Somsouk, Jennifer C Lai, Ki Lai, Jin Ge

**Affiliations:** Division of Gastroenterology and Hepatology, Department of Medicine at the University of California, San Francisco, San Francisco, CA, United States; UCSF Health Information Technology at the University of California, San Francisco, San Francisco, CA, United States; Division of Gastroenterology and Hepatology, Department of Medicine at the University of California, San Francisco, San Francisco, CA, United States; Division of Gastroenterology and Hepatology, Department of Medicine at the University of California, San Francisco, San Francisco, CA, United States; Division of Gastroenterology and Hepatology, Department of Medicine at the University of California, San Francisco, San Francisco, CA, United States; UCSF Health Information Technology at the University of California, San Francisco, San Francisco, CA, United States; UCSF Health Information Technology at the University of California, San Francisco, San Francisco, CA, United States; Division of Gastroenterology and Hepatology, Department of Medicine at the University of California, San Francisco, San Francisco, CA, United States; Division of Gastroenterology and Hepatology, Department of Medicine at the University of California, San Francisco, San Francisco, CA, United States; UCSF Health Information Technology at the University of California, San Francisco, San Francisco, CA, United States; Division of Gastroenterology and Hepatology, Department of Medicine at the University of California, San Francisco, San Francisco, CA, United States; Bakar Computational Health Sciences Institute at the University of California, San Francisco, San Francisco, CA, United States

**Keywords:** Large language models, generative artificial intelligence, gastroenterology, liver diseases, data analysis

## Abstract

**Objective:**

Large language models (LLMs) may improve analysis of structured clinical data by enabling natural language (NL) queries. We developed an interactive, PHI-compliant “Text2SQL” chatbot as a proof-of-concept for hepatocellular carcinoma (HCC) research.

**Materials and methods:**

Using the University of California, San Francisco’s secure artificial intelligence platform, we configured the chatbot to translate NL queries into Structured Query Language (SQL), execute them, and return both code and results. Clinicians posed 30 questions, and outputs were compared with SQL written by a data scientist. We assessed prompt interpretation, SQL accuracy, output accuracy, and error types according to strict binary criteria.

**Results:**

Prompt interpretation accuracy was 73.3%, SQL accuracy ranged from 53% to 63%, and output accuracy from 53% to 63%. Frequent errors included group comparisons, ambiguous variables, and exploratory questions.

**Discussion:**

Text2SQL enables PHI-compliant interaction with clinical datasets without coding, though performance varies across query types.

**Conclusion:**

LLM-powered Text2SQL offers a promising approach for clinician-friendly clinical data access.

## Background and significance

Accessing insights from clinical databases often requires familiarity with Structured Query Language (SQL), limiting healthcare professionals without coding expertise from utilizing structured clinical research datasets. Large language models (LLMs) enable interaction with data through natural language, allowing users to pose free-text questions that are converted into executable queries. Commercial LLMs, such as OpenAI’s ChatGPT, demonstrate this capability, but their use raises concerns for protected health information (PHI).

Prior work has advanced text-to-SQL systems using benchmark datasets (e.g., Spider, BIRD).^1^^,^^2^ Clinical efforts such as MedT5SQL and EHRSQL evaluate reliable SQL generation for electronic health records.^3^^,^^4^ However, few studies address deployment in live, protected health information (PHI) protected clinical settings, where secure architecture, iterative error handling, and scalable design are essential for real-world use.

HIPAA-eligible LLM services, such as Azure OpenAI, and the University of California San Francisco (UCSF)’s secure Generative AI (GenAI) platform, Versa, provide frameworks for PHI-safe deployment. As health systems increasingly adopt PHI-compliant LLMs, there is a growing need for PHI-compliant systems that can safely bridge the gap between NL interfaces and clinical databases. To address this, we developed and deployed a secure “Text2SQL” system within Versa.

## Objective

In this validation study, we tested Text2SQL on a dataset of hepatocellular carcinoma (HCC) and frailty in patients evaluated for liver transplantation. We aimed to assess the performance of this PHI-compliant, LLM-driven system to enable secure, free-text querying of **this** clinical dataset.

## Materials and methods

### Dataset

We used the “HCC-frailty” dataset from UCSF, which includes 2420 patients with HCC enrolled in the multicenter Functional Assessment in Liver Transplantation (FraILT) at UCSF.^5^ The dataset contains one table with structured variables on demographics, comorbidities, laboratory values, imaging findings, treatment history, and frailty-related indicators. Use of the database for this exploratory exercise is approved under UCSF Institutional Review Board under Study #11-07513.

To enable secure access from the Versa platform, the dataset was first placed within a Python Simple Container-Utilizing Build Apparatus (SCUBA), a tool to create and manage a containerized development environment.^6^ SCUBA, enabling version control of software libraries, ensured that software updates within the Versa platform did not affect the development of Text2SQL.

### Text2SQL system setup

The architecture for our Text2SQL system follows a shared-responsibility model: the LLM service provides HIPAA-eligible model hosting, while UCSF ensures proper access controls, logging, network isolation, and PHI governance. If deployed elsewhere, the system’s compliance would depend on similar infrastructure.

**Figure 1.**
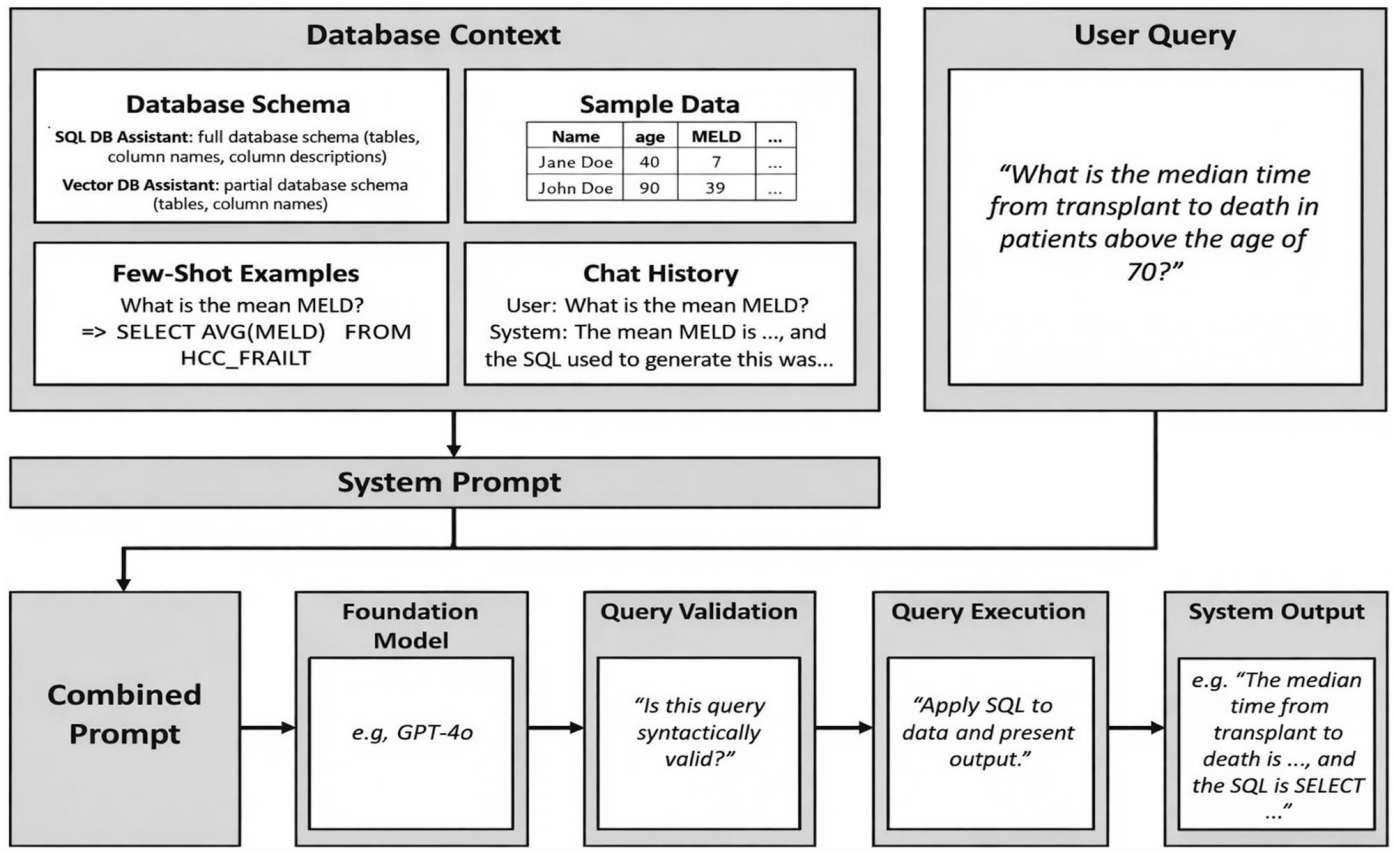
“Text2SQL” overall configuration and pipeline.

To generate accurate and meaningful SQL, the Text2SQL architecture uses multiple layers of information. The system composes these layers into a prompt, which is sent to an internally hosted LLM endpoint within Versa using Chat Completions API^7^ to generate the SQL query. The system performs query validation for verifying SQL syntax, using the Tool Use Design Pattern^8^ to iteratively run the query with the Microsoft Azure SQL Database engine (Transact-SQL dialect; version 12.0.2000.8) engine and provide errors back to the model as needed. It executes this validated query on the dataset and provides the output (Figure 1). The layers of contextual data that the system uses include the following:

1. Database Schema: Critical structural knowledge such as table names, column names, data types, and key relationships, ensuring that the generated SQL aligns with the actual structure of the target database.
2. Sample Data: Representative rows from tables help the system infer data formats, ranges, and categorical values.
3. Few-shot Examples: Curated examples of user prompts and correct SQL outputs fed into the LLM to provide examples of task format and expectations.
4. Chat History: Prior interactions within the chat session, including questions, model responses, and query results, to maintain context and enable users to reference earlier outputs or add new constraints without restating the prompt.

**Figure 2.**
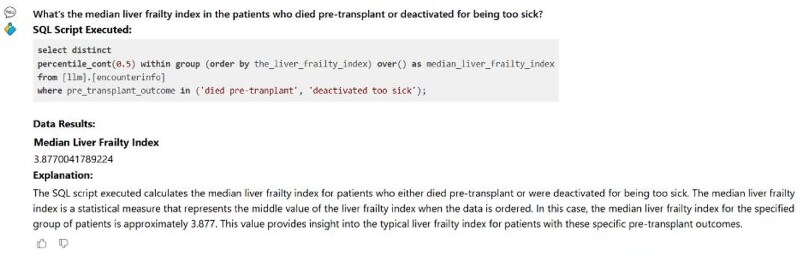
“Text2SQL” example of chat interface.

This multi-source context forms a comprehensive system prompt that enables the LLM to produce SQL from raw user input that is both syntactically valid and semantically appropriate. The system returns both the query results and an accompanying explanation through the same interface, allowing data interaction without coding (Figure 2).

**Figure 3.**
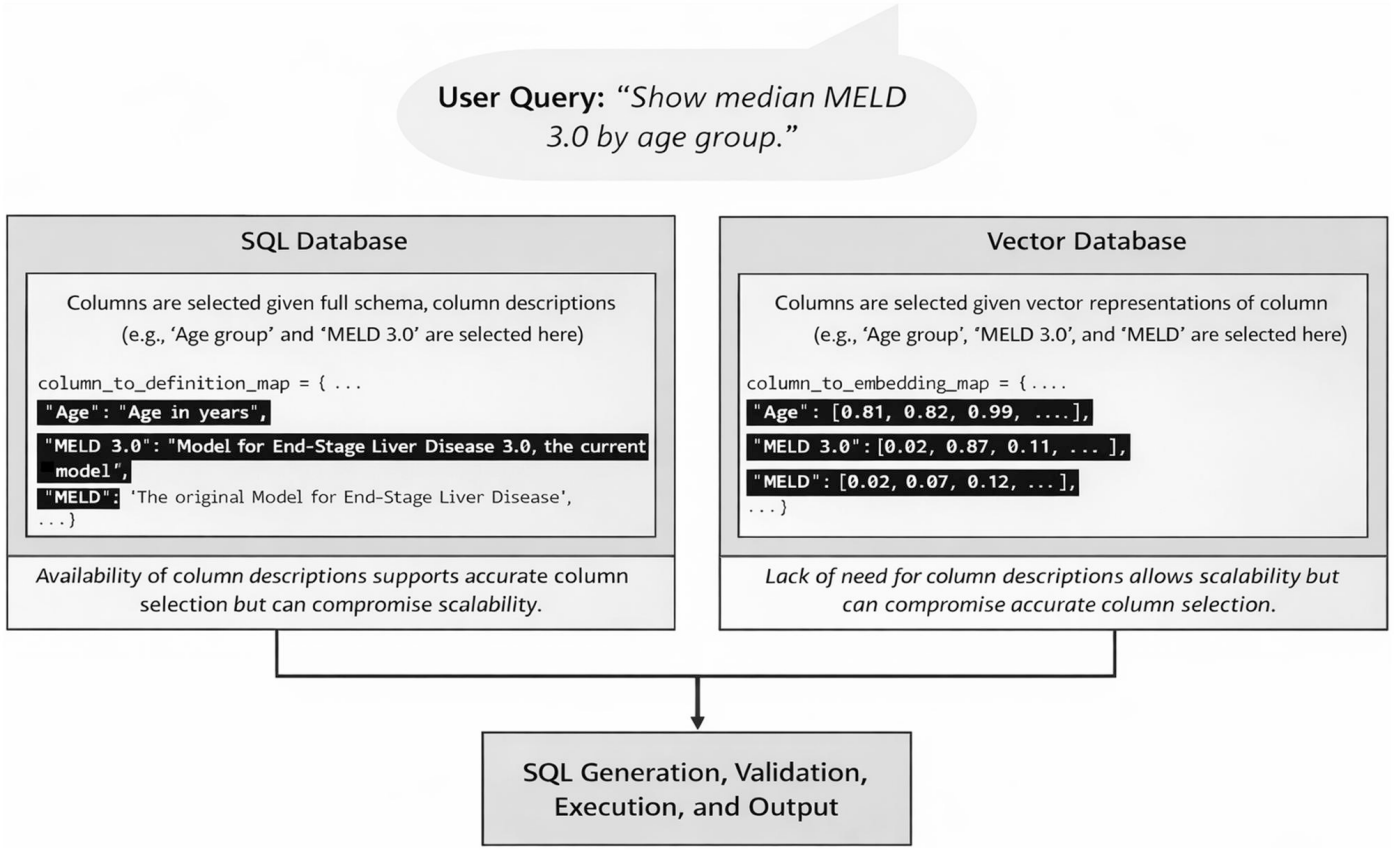
Comparison of SQL database versus vector database in data processing.

**Figure 4.**
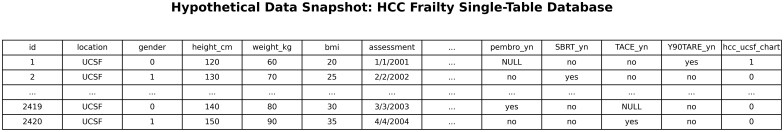
Hypothetical data snapshot.

Two versions of this Text2SQL system were built, each with different underlying column look-up mechanisms. The first version, SQL Database (DB), shares the full database schema, including descriptions for all columns, with the LLM. The second version, Vector DB, does not use column descriptions. Instead, it performs a semantic search over vectorized representations of column names and categorical column values (e.g., column “reason_withdrawn_from_study” with values “Administrative withdrawal,” “Patient withdrew consent,” “Other”) to identify the top relevant columns based on the user’s question. This system version leverages 1536-dimensional embeddings via the text-embedding-ada-002 model^9^ to search for columns through the cosine similarity metric, with no threshold. Once relevant columns are retrieved, a streamlined schema is dynamically constructed and passed to the model (Figure 3). This approach is designed to scale to larger or multi-table databases, as it does not rely on column descriptions. However, we tested it only on a single-table dataset (Figure 4).

Due to its prompting strategy, Text2SQL could be adapted for any foundation LLM within our PHI-protected platform. We tested the system using Microsoft Azure OpenAI’s Generative Pre-trained Transformer 4-Omni (GPT-4o).^10^

### Evaluation approach

To assess Text2SQL’s performance, we tested the system using 30 free-form questions about the HCC-frailty dataset, drafted by team members who worked frequently with the dataset (G.J. and M.W.). Team members were free to draft any clinical research questions relevant to the dataset. These queries covered a range of question types, including retrieving specific values, filtering results, grouping, listing records based on specified criteria, and other exploratory questions. For a list of the questions posed, see Supplemental Materials, available as supplementary material  *[JAMIA Open]* online.

Each query was submitted through the Text2SQL chatbot interface. The system-generated SQL and corresponding outputs were recorded. To evaluate accuracy, we compared system outputs against ground truth responses created manually by a data scientist (A.T.F) using Python and SQL. We assessed Text2SQL results by the following binary criteria with predefined tolerances: (1) prompt interpretation accuracy, (2) SQL accuracy, defined as whether the query matched the user’s intended prompt logic, and (3) output validity, defined as whether the result set matched ground truth within specified tolerances. Tolerances were applied to numeric and date values with accurate SQL code (off by at most 1% for numeric aggregates) to accommodate small deviations arising from SQL engine rounding, data type handling, or LLM-mediated query execution, allowing outputs to be scored consistently. We calculated 95% confidence intervals (CI) for accuracies using the Clopper-Pearson method. Both system versions, SQL DB and Vector DB, were evaluated using the same set of questions. We qualitatively compared their performance on SQL accuracy and output validity. Observations were also made regarding system usability and specific error types.

## Results

### Performance

The SQL DB assistant achieved 73.3% (95% confidence interval [CI] 54.1%-87.7%) accuracy for prompt interpretation, 63.3% (95%CI 44.9%-79.7%) accuracy for SQL code, and 63.3% (95%CI 44.9%-79.7%) accuracy for output. The Vector DB assistant achieved 60% (95%CI 40.6%-77.3%) accuracy for prompt interpretation, 53.3% (95%CI 34.3%-71.7%) accuracy for SQL code, and 53.3% (95%CI 34.3%-71.7%) accuracy for output. The confidence intervals for the two systems overlap across all evaluation metrics, and observed differences in point estimates were modest. See Table 1 for these side-by-side results.

**Table 1.** Side-by-side system version accuracies.

Metric	SQL Version	Vector DB Version
Prompt interpretation accuracy (95% CI)	73.3% (54.1%-87.7%)	60% (40.6%-77.3%)
SQL code accuracy (95% CI)	63.3% (44.9%-79.7%)	53.3% (34.3%-71.7%)
Output accuracy (95% CI)	63.3% (44.9%-79.7%)	53.3% (34.3%-71.7%)

### Error analysis

Both system versions reliably handled simple queries, such as identifying common diagnoses, calculating median MELD scores, or counting patients who met specific criteria. However, performance diverged on more complex or ambiguous prompts. The SQL DB version generally performed better on stratified comparisons and multi-condition logic. For example, in response to “Provide a table comparing patients over 70 vs under 70: median LFI and MELD 3.0.”

**SQL DB:** SELECT CASE WHEN Age > 70 THEN 'Over 70' ELSE 'Under 70' END AS Age_Group, PERCENTILE_CONT(0.5) WITHIN GROUP (ORDER BY The_Liver_Frailty_Index) OVER(PARTITION BY CASE WHEN Age > 70 THEN 'Over 70' ELSE 'Under 70' END) AS Median_LFI, PERCENTILE_CONT(0.5) WITHIN GROUP (ORDER BY meld_pre_last) OVER(PARTITION BY CASE WHEN Age > 70 THEN 'Over 70' ELSE 'Under 70' END) AS Median_MELD_3_0 FROM [llm].[TransplantInfo];

**Vector DB:** SELECT CASE WHEN Age > 70 THEN ‘Over 70’ ELSE ‘Under 70’ END AS Age_Group, PERCENTILE_CONT(0.5) WITHIN GROUP (ORDER BY The_Liver_Frailty_Index) OVER(PARTITION BY CASE WHEN Age > 70 THEN ‘Over 70’ ELSE ‘Under 70’ END) AS Median_LFI, PERCENTILE_CONT(0.5) WITHIN GROUP (ORDER BY MELD_at_Transplant) OVER(PARTITION BY CASE WHEN Age > 70 THEN ‘Over 70’ ELSE ‘Under 70’ END) AS Median_MELD FROM [llm].[TransplantInfo] GROUP BY CASE WHEN Age > 70 THEN ‘Over 70’ ELSE ‘Under 70’ END;

SQL DB used correct stratification logic via PARTITION BY, returning a valid result. Vector DB, although it recognized the stratification intent, mistakenly added a GROUP BY, preventing valid output. This pattern was reflected across error rates for group comparisons (SQL DB: 1 error, Vector DB: 4) and output failures (SQL DB: 2, Vector DB: 7).

Both versions struggled with exploratory queries requiring creative column usage. For example, “What is the most common reason for delisting?”

**SQL DB:** SELECT Pre_transplant_Outcome, COUNT(*) AS Count FROM [llm].[TransplantInfo] WHERE Pre_transplant_Outcome LIKE 'Delisted%' GROUP BY Pre_transplant_Outcome ORDER BY Count DESC

The system returned a query that counted delisted patients but did not extract the actual reasons for delisting. While syntactically valid, this output was only tangentially related to the question. This highlights the system’s current inability to effectively combine information from related columns that could provide insight into the reasons for delisting.

Another failure involved “What are HCC tumor characteristics in patients over 70 listed for transplant?” Both versions attempted to retrieve this information but failed.

**SQL DB:** SELECT COUNT(*) AS Number_of_Tumors, PERCENTILE_CONT(0.5) WITHIN GROUP (ORDER BY Tumor_Size) OVER() AS Median_Tumor_Size FROM [llm].[TransplantInfo] WHERE Age > 70 AND HCC = 1;

**Vector DB:** SELECT COUNT(*) AS Number_of_Tumors, PERCENTILE_CONT(0.5) WITHIN GROUP (ORDER BY HCC_at_Transplant) OVER() AS Median_Tumor_Size FROM [llm].[TransplantInfo] WHERE Age > 70 AND HCC = 1;

SQL DB attempted to calculate a median Tumor_Size, but may have encountered missing values in that column. Vector DB used HCC_at_Transplant, a non-numeric indicator, within a percentile function. In both cases, the queries executed but returned nothing, indicating a failure to handle sparse data and a lack of error resilience to incorrect assumptions about column structure.

Column ambiguity challenged both systems. For example: “What is the median LFI and MELD 3.0 in patients over 70 listed for transplant?”

**SQL DB:** SELECT PERCENTILE_CONT(0.5) WITHIN GROUP (ORDER BY The_Liver_Frailty_Index) OVER() AS Median_LFI, PERCENTILE_CONT(0.5) WITHIN GROUP (ORDER BY meld_pre_last) OVER() AS Median_MELD_3_0 FROM [llm].[TransplantInfo] WHERE Age > 70;

**Vector DB:** SELECT PERCENTILE_CONT(0.5) WITHIN GROUP (ORDER BY The_Liver_Frailty_Index) OVER() AS Median_LFI, PERCENTILE_CONT(0.5) WITHIN GROUP (ORDER BY MELD_at_Transplant) OVER() AS Median_MELD FROM [llm].[TransplantInfo] WHERE Age > 70;

SQL DB correctly used meld_pre_last, representing MELD before transplant evaluation. Vector DB used MELD_at_Transplant, which reflects MELD at transplant. Given the query referred to patients “listed for transplant,” MELD prior to evaluation is more appropriate. This example highlights difficulty in deductive column selection based on clinical context as well as column name similarity, with high errors for incorrect column usage (SQL DB: 5, Vector DB: 7).

Overall, SQL DB produced more correct completions (19 vs 16) and had fewer errors, suggesting greater robustness in handling queries. Error frequencies by category and system version are in Table 2.

**Table 2.** Side-by-side error analysis comparison.

Error Category	SQL DB Version	Vector DB Version
Incorrect column selection (e.g., wrong date, MELD timing)	5	7
Incorrect group comparison	1	4
Failure to retrieve output	2	7
Misinterpretation of ambiguous prompt	4	4
Error in numeric output beyond tolerance	1	1
Prompt interpretation failure	2	2
SQL generation failure or hallucination	1	3
Errors in statistical logic or calculation	1	1
Fully correct (no errors)	19	16

## Discussion

Our implementation demonstrates the feasibility of using LLMs for clinical data querying within a secure, PHI-compliant environment. By integrating a Text2SQL system into UCSF’s Versa platform, we enabled users to retrieve structured clinical data through NL alone without code. This no-code interface greatly lowers the barrier to data access for non-technical users, offering an intuitive way to interact with complex datasets. In addition to a schema-based version, SQL DB, we developed a vector-based version, Vector DB, that dynamically selects relevant columns based on semantic similarity.

In this single-table context, SQL DB had higher point estimates of accuracy than Vector DB, possibly due to access to full schema descriptions. Vector DB’s use of embeddings could lead to selecting semantically similar but incorrect column selection. However, Vector DB is designed for scalability, which may benefit larger or multi-table databases where detailed schemas are hard to maintain. This may reflect an accuracy and scalability tradeoff, with Vector DB offering flexibility at some cost to performance.

We also observed limitations. Although the Vector DB approach was designed to scale to multi-table environments, our evaluation was limited to a single-table dataset, so its performance in more complex schemas remains unknown. Both versions faced challenges when user intent did not align well with naming conventions. The system was not well-suited to exploratory analyses and performed best with specific, well-formed questions, consistent with recent work demonstrating the advantages of using predefined clinical queries.^11^ Furthermore, system performance may vary depending on the foundation model: OpenAI’s o1 model excels in complex problem-solving and handling ambiguous queries,^12^ while Anthropic’s Claude models demonstrate strength in coding and long-context reasoning, ^13^^,^^14^ potentially favoring longer and more structured queries. Finally, because our questions were authored by individuals familiar with this dataset, the evaluation reflects typical research use but may not generalize to users without prior schema exposure.

Future work could explore template-guided SQL generation (e.g., using prompt templates and fragments like AlloyDB),^15^ as well as commercial NL-to-SQL tools such as DBCopilot^16^ or ODIN, which generate multiple candidate queries to resolve ambiguity.^17^ Refining prompts, adding diverse few-shot examples, and developing an interactive clarification feature (e.g., system asks user, “Do you mean MELD score at transplant or at listing?”) may improve accuracy and usability. Testing the system on multi-table databases would provide important insight into its scalability and effectiveness in more complex settings. Future phases should also include a broader range of users to assess generalizability.

## Conclusion

This study shows that systems like Text2SQL can support PHI-protected clinical data access when questions are clear and variables are well-defined. Overall, these findings provide insight into how LLM-driven querying behaves when used with typical clinical research queries.

## Supplementary Material

ooag106_Supplementary_Data

## Data Availability

De-identified aggregated data used for the motivating example in the manuscript could be made available upon reasonable request to the authors.
