# Supplementary material for: Large language model chatbot-based text-to-SQL application for database analyses in liver diseases and hepatology research
Source: JAMIA Open. 2026 Jun 19;9(3):ooag106. doi: 10.1093/jamiaopen/ooag106 (PMC13278831; doi:10.1093/jamiaopen/ooag106)
Supplement: ooag106_Supplementary_Data [file ooag106_supplementary_data.docx]

**SUPPLEMENTARY MATERIALS**

**Queries Written by Researchers and Performances**

1. What is the most common post-transplant complication?
   1. Prompt interpretation: SQL DB **incorrect**; Vector DB **incorrect**.
   2. SQL code: SQL DB **incorrect**; Vector DB **incorrect**.
   3. Output: SQL DB **incorrect**; Vector DB **incorrect**.
2. What is the most common liver disease etiology among candidates with an LFI > 4.0?
   1. Prompt interpretation: SQL DB correct; Vector DB correct.
   2. SQL code: SQL DB correct; Vector DB correct.
   3. Output: SQL DB correct; Vector DB correct.
3. What percentage of patients with LFI > 4.0 are older than 65 years?
   1. Prompt interpretation: SQL DB correct; Vector DB correct.
   2. SQL code: SQL DB correct; Vector DB correct.
   3. Output: SQL DB correct; Vector DB correct.
4. What is the median wait time for transplant from the time of listing among patients with LFI > 4.5?
   1. Prompt interpretation: SQL DB correct; Vector DB correct.
   2. SQL code: SQL DB correct; Vector DB correct.
   3. Output: SQL DB correct; Vector DB correct.
5. What is the median LFI for patients with HCC compared to those without HCC?
   1. Prompt interpretation: SQL DB correct; Vector DB **incorrect**.
   2. SQL code: SQL DB correct; Vector DB **incorrect**.
   3. Output: SQL DB correct; Vector DB **incorrect**.
6. How many patients underwent simultaneous kidney and liver transplant?
   1. Prompt interpretation: SQL DB correct; Vector DB correct.
   2. SQL code: SQL DB correct; Vector DB correct.
   3. Output: SQL DB correct; Vector DB correct.
7. What is the 6-month and 1-year post-transplant survival of patients who underwent SLK?
   1. Prompt interpretation: SQL DB correct; Vector DB correct
   2. SQL code: SQL DB correct; Vector DB correct
   3. Output: SQL DB correct; Vector DB correct
8. What is the median MELD at transplant of patients with liver disease related to alcohol?
   1. Prompt interpretation: SQL DB correct; Vector DB correct
   2. SQL code: SQL DB correct; Vector DB correct
   3. Output: SQL DB correct; Vector DB correct
9. Which blood type is the most common among pre-transplant patients?
   1. Prompt interpretation: SQL DB correct; Vector DB correct
   2. SQL code: SQL DB correct; Vector DB correct
   3. Output: SQL DB correct; Vector DB correct
10. What percentage of patients with CAD ultimately get transplanted?
    1. Prompt interpretation: SQL DB correct; Vector DB correct
    2. SQL code: SQL DB correct; Vector DB correct
    3. Output: SQL DB correct; Vector DB correct
11. How many patients have abnormal kidney function with eGFR < 60 at the time of transplant?
    1. Prompt interpretation: SQL DB **incorrect**; Vector DB **incorrect**
    2. SQL code: SQL DB **incorrect**; Vector DB **incorrect**
    3. Output: SQL DB **incorrect**; Vector DB **incorrect**
12. What is the proportion of patients with diabetes, CAD, and hypertension who ultimately get transplanted?
    1. Prompt interpretation: SQL DB correct; Vector DB correct
    2. SQL code: SQL DB correct; Vector DB correct
    3. Output: SQL DB correct; Vector DB correct
13. What is the mean LFI of patients 70 years or older who are transplanted?
    1. Prompt interpretation: SQL DB correct; Vector DB correct
    2. SQL code: SQL DB correct; Vector DB correct.
    3. Output: SQL DB correct; Vector DB correct.
14. What is the median LFI of patients with ascites compared to those without ascites?
    1. Prompt interpretation: SQL DB correct; Vector DB correct.
    2. SQL code: SQL DB correct; Vector DB correct
    3. Output: SQL DB correct; Vector DB correct
15. What is the most common reason for delisting?
    1. Prompt interpretation: SQL DB **incorrect**; Vector DB **incorrect**.
    2. SQL code: SQL DB **incorrect**; Vector DB **incorrect**.
    3. Output: SQL DB **incorrect**; Vector DB **incorrect**.
16. How many patients over 70 years old underwent deceased donor liver transplantation?
    1. Prompt interpretation: SQL DB correct; Vector DB correct.
    2. SQL code: SQL DB **incorrect**; Vector DB correct.
    3. Output: SQL DB **incorrect**; Vector DB correct.
17. What is the breakdown of liver disease types in patients over 70 listed for transplant?
    1. Prompt interpretation: SQL DB correct; Vector DB correct
    2. SQL code: SQL DB correct; Vector DB correct
    3. Output: SQL DB correct; Vector DB correct
18. What is the median LFI and MELD 3.0 in patients over 70 listed for transplant?
    1. Prompt interpretation: SQL DB correct; Vector DB **incorrect**.
    2. SQL code: SQL DB correct; Vector DB **incorrect**.
    3. Output: SQL DB correct; Vector DB **incorrect**.
19. Provide a table comparing patients over 70 vs under 70: median LFI and MELD 3.0.
    1. Prompt interpretation: SQL DB correct; Vector DB **incorrect**.
    2. SQL code: SQL DB correct; Vector DB **incorrect**
    3. Output: SQL DB correct; Vector DB **incorrect**.
20. How many patients over 70 listed for transplant had HCC?
    1. Prompt interpretation: SQL DB correct; Vector DB correct.
    2. SQL code: SQL DB correct; Vector DB correct.
    3. Output: SQL DB correct; Vector DB correct.
21. What are HCC tumor characteristics in patients over 70 listed for transplant?
    1. Prompt interpretation: SQL DB **incorrect**; Vector DB **incorrect**.
    2. SQL code: SQL DB **incorrect**; Vector DB **incorrect**.
    3. Output: SQL DB **incorrect**; Vector DB **incorrect**.
22. Compare median creatinine in patients over 70 vs under 70.
    1. Prompt interpretation: SQL DB correct; Vector DB correct.
    2. SQL code: SQL DB correct; Vector DB **incorrect**.
    3. Output: SQL DB correct; Vector DB **incorrect**.
23. Describe HRQOL metrics in patients over 70 listed for transplant.
    1. Prompt interpretation: SQL DB **incorrect**; Vector DB **incorrect**.
    2. SQL code: SQL DB **incorrect**; Vector DB **incorrect**.
    3. Output: SQL DB **incorrect**; Vector DB **incorrect**.
24. Summarize HRQOL data with binary counts and continuous medians (IQR).
    1. Prompt interpretation: SQL DB **incorrect**; Vector DB **incorrect**.
    2. SQL code: SQL DB **incorrect**; Vector DB **incorrect**.
    3. Output: SQL DB **incorrect**; Vector DB **incorrect**.
25. What is the 1-year, 3-year, and 5-year post-transplant survival in patients over 70 (DDLT)?
    1. Prompt interpretation: SQL DB correct; Vector DB correct.
    2. SQL code: SQL DB **incorrect**; Vector DB correct.
    3. Output: SQL DB **incorrect**; Vector DB correct.
26. What is the median survival in months for patients over 70 undergoing DDLT?
    1. Prompt interpretation: SQL DB **incorrect**; Vector DB **incorrect**.
    2. SQL code: SQL DB **incorrect**; Vector DB **incorrect**.
    3. Output: SQL DB **incorrect**; Vector DB **incorrect**.
27. What are the 1-month, 3-month, and 6-month survival rates in patients over 70 undergoing DDLT?
    1. Prompt interpretation: SQL DB correct; Vector DB correct.
    2. SQL code: SQL DB **incorrect**; Vector DB correct.
    3. Output: SQL DB **incorrect**; Vector DB correct.
28. What is the median ICU and hospital length of stay in patients over 70 undergoing DDLT?
    1. Prompt interpretation: SQL DB correct; Vector DB correct**.**
    2. SQL code: SQL DB correct; Vector DB **incorrect**.
    3. Output: SQL DB correct; Vector DB **incorrect**.
29. Is the median LFI statistically significantly higher in patients with LOS >6 days vs <6 days (age >70)?
    1. Prompt interpretation: SQL DB **incorrect**; Vector DB **incorrect**.
    2. SQL code: SQL DB **incorrect**; Vector DB **incorrect**.
    3. Output: SQL DB **incorrect**; Vector DB **incorrect**.
30. Provide median LFI in patients with LOS >6 days vs <6 days (age >70).
    1. Prompt interpretation: SQL DB correct; Vector DB **incorrect**.
    2. SQL code: SQL DB correct; Vector DB **incorrect**.
    3. Output: SQL DB correct; Vector DB **incorrect**

**Side-by-side Error Analysis Comparison with Question Numbers**

| **Error Category** | **SQL DB Version (number of errors: question #s)** | **Vector DB Version**  **(number of errors: question #s)** |
| --- | --- | --- |
| Incorrect column selection (e.g., wrong date, MELD timing) | 5:  #11, 16, 21, 25, 26 | 7:  #11, 18, 19, 21, 26, 28, 30 |
| Incorrect group comparison | 1:  #27 | 4:  #5, 18, 19, 22 |
| Failure to retrieve output | 2:  #21, 24 | 7:  #5, 18, 19, 21, 22, 24, 29 |
| Misinterpretation of ambiguous prompt | 4:  #21, 23, 24, 29 | 4:  #21, 23, 24, 29 |
| Error in numeric output beyond tolerance | 1:  #1 | 1:  #1 |
| Prompt interpretation failure | 2:  #1, 11 | 2:  #1, 11 |
| SQL generation failure or hallucination | 1:  #27 | 3:  #21, 22, 30 |
| Errors in statistical logic or calculation | 1:  #29 | 1:  #29 |
| Fully correct (no errors) | 19:  #2, 3, 4, 5, 6, 7, 8, 9, 10, 12, 13, 14, 17, 18, 19, 20, 22, 28, 30 | 16:  #2, 3, 4, 6, 7, 8, 9, 10, 12, 13, 14,  16, 17, 20, 25, 27 |
